# The relationship of Value dimensions in Turk Society with fatalistic tendencies, safety motivation, risk perception and safety performance

**DOI:** 10.1016/j.heliyon.2024.e30384

**Published:** 2024-04-26

**Authors:** Ahmet Ebrar Sakalli, Selma Arikan

**Affiliations:** a Department of Occupational Health and Safety, Istanbul Aydın University, İstanbul, Turkiye; b Department of Psychology, Istanbul Medeniyet University, İstanbul, Turkiye

**Keywords:** Values, Fatalistic tendencies, Risk perception, Safety motivation, Safety performance

## Abstract

Understanding the role of people in occupational accidents is very difficult. It is always assumed that people perform the behaviour that feels right to them. This study's main topic is the relationship between values, which form the basis of human behaviour, and safety motivation, fatalistic tendencies, risk perception and safety behaviour.

In this context, for the aim of the study, path analysis and partial correlation analysis were used to examine the relationship between variables, and tests were used to examine the relationship between demographic variables with 701 participants from NUTS-12 region of Turkey.

According to the findings of the research, it was determined that most of the 19 value dimensions and the top values of self-protection and growth and the top value dimensions of conservation, self-enhancement, self-transcendence and openness to change affect fatalism tendencies, risk perception, safety motivation(SM) and safety performance(SP). While self-protection, conservation and self-enhancement top values have a negative effect on SP, openness to change, self-transcendence and growth top values have a positive effect on SP.

Taking into consideration that individuals will exhibit behaviours based on the value dimensions they attach importance to, OHS trainings should be developed in accordance with the value dimensions given importance according to NUTS-12 regions. Furthermore, legal support should be provided to eliminate and reduce the negative aspects of value dimensions for OHS.

## 1 Introduction

Many studies conducted in different countries and in different sectors reveal that the main cause of accidents is human error-behaviour [[1], [2], [3], [4], [5], [6], [7], [8], [9], [10], [11], [12]]. National culture has long been recognized as important in explaining behaviour (Ng, Lee & Soutar 2007). The complex and wide nature of culture makes it difficult to measure accurately. This difficulty can pose some problems for determining the impact of culture on behaviour. Researchers recommend global indices or individual level reports to simplify operations and make certain aspects of culture easier to apply [13]. Culture, which emerges as a result of attitudes and behaviours, reflects how employees act and how they are treated [14].

The study focuses on values and its implications on fatalistic tendencies, safety motivation, risk perception and safety performance which are explained in next sub-sections.

### 1.1 Safety culture

To better grasp the meaning of safety culture, there is a need to go back to its origins [15,16]. According to a sociological definition, culture consists of the values held by a particular group of members, the norms they follow and the material elements they create [17].

Values and norms are transmitted through the process of socialization, as they are learned by people in groups. Therefore, culture is also a phenomenon learned through socialization. Safety culture can be understood as an analytical concept, not an empirical entity [18]. Thus, safety culture is a label that represents the relationship between culture and safety and is not a separate entity in its own right [19]. Giddens [17], in line with his definition of culture Mearns ve Flin [20], safety culture as a complex and enduring characteristic of a workplace that reflects the core values, norms, assumptions and expectations found to some extent in societal culture.

In recent years, researchers have paid great attention to the role of cultural factors as antecedents of accident occurrences [[21], [22], [23], [24], [25], [26]]. In Türkiye, there are studies indicating that safety culture is quite weak and that the main causes of accidents are these weaknesses in safety culture [8].

**Fig. 1:**
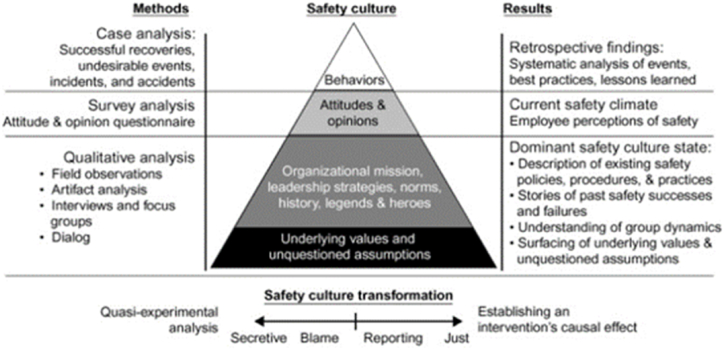
Pyramid of safety culture [27].

Patankar and Sabin (2010: 99–101), examined the structure of safety culture in an organization as a pyramid consisting of four steps (Fig. 1). At the bottom of the pyramid are core safety values and unquestioned assumptions. At the top are organizational factors, leadership strategies, norms, history, legends and heroes. Above this step are attitudes and opinions (safety climate), while at the top of the pyramid are safety behaviours (SP).

As can be seen in Fig. 1, several studies have been conducted on safety culture and safety climate, which are two different concepts. Safety culture can be defined as the beliefs, values, attitudes and behaviour patterns that a group of people share about safety [28]. There are numerous studies showing that attitudes and behaviours are significantly related [29]. Fig. 1 shows that attitudes and opinions (safety climate) affect safety behaviours (SP).

### 1.2 Definition of values

There are various factors that affect people's perceptions, attitudes, and behaviours, including beliefs, norms and etc. One of these factors is the values of the individual. Values guide and help individuals in evaluating events and behaviours [30]. People prefer one behaviour over others in line with their values [31]. Values are "intangible ideals", perceptions of what is right and wrong and how things should and should not be [17]. Values are important tools used to distinguish cultural groups, societies and individuals. Values also play an important role in tracking changes over time and explaining the motivational bases of attitudes and behaviours (Schwartz, 2012).

Many studies suggest that values constitute the most important aspect of culture[32,33]. Values are one of the most important and intensively studied concepts in social-psychological research in relation to cultures. Research shows that cultural characteristics are manifested in the evaluation of individuals' priorities and that these evaluations reveal the basic values that individuals use to classify different behaviours and situations as right or wrong, doable or undoable, desirable or undesirable [[34], [35], [36]].

The performance of a behaviour is a result of factors such as opportunities, constraints, ability and motivation. Values, as motivators, guide our behaviour by reflecting desired states. Therefore, our values have a great influence behind our behaviour [37].

To affect behaviour, values must first be activated, experienced consciously or through external awareness of aspects of a situation. Since values that are more important to us are more accessible than others, they are more likely to be activated and influence behaviour [38]. Studies showing stronger associations between value priorities and behaviour provide evidence of the causal effect of values [39,40].

### 1.3 Shalom H. Schwartz's theory of values

At the individual level, value priorities refer to the alignment an individual makes to pursue a particular value. In this case, participants tend to emphasize values that are important to them, while downplaying less important or conflicting values [36].

Since values are based on basic motivations, they can be compatible or in conflict with each other. For instance, an individual who values achievement may conflict with the value of benevolence. This is because one cannot always consider the well-being of others when pursuing one's own desires. However, the same individual may harmoniously embrace the value of power and follow a path in accordance with it [41].

Schwartz et al. [42], revisited the theory of values with data from ten countries, including Türkiye, and identified nineteen value types that are more descriptive than ten value types. Table 1 shows the nineteen values reviewed and their brief definitions.

**Table 1:** Schwartz's values and definitions.

	Value	Definition
1	**Self-direction-thought**	Freedom to develop one's own thoughts and abilities
2	**Self-direction-action**	Freedom to decide one's own actions
3	**Stimulation**	Excitement, search for novelty, desire for change
4	**Hedonism**	Pleasure and satisfaction of the senses
5	**Achievement**	Being successful according to societal criteria
6	**Power-dominance**	It refers to the power gained through control over people
7	**Power-resources**	It expresses the power gained through control over societal resources and substances
8	**Face**	It expresses the security and power one achieves by avoiding humiliation and maintaining one's societal image
9	**Security- personal**	The trustworthiness of one's close circle
10	**Security-societal**	It stands for the trustworthiness and stability of society
11	**Tradition**	It expresses the preservation and maintenance of cultural, familial and religious traditions
12	**Conformity-rules**	It refers to compliance with rules, laws and legal obligations
13	**Conformity-interpersonal**	It expresses refraining from upsetting or harming other individuals
14	**Humility**	It expresses a person's undervaluation of certain things
15	**Benevolence-dependability**	It refers to being a reliable and believable member of an internal cluster (group)
16	**Benevolence - caring**	It expresses selfless behaviour for the good of the inner cluster members
17	**Universalism-concern**	Ensuring equality and justice refers to the protection of all other people
18	**Universalism-nature**	Refers to protecting the natural environment.
**19**	Universalism-tolerance	**It stands for understanding and accepting those who are different from one's own self**

*Source:* [42].

In explaining the dynamics of values, Schwartz mentions the existence of values with similar motives as well as conflicting values. According to the theory, values do not have precise lines, but the difference in basic motives between some value groups allows for a rational and realistic distinction. In this context, Schwartz categorized values under two super headings, namely Self-Enhancement - Self Transcendence and Openness to Change - Conservation. These headings include values that are expected to be in competition with each other [43].

Self Enhancement - Self Transcendence: This meta-heading involves the conflict between achievement/power (self-aggrandizement/self-enhancement) values and universalism/benevolence (self-transcendence) values. Schwartz states that Self-Enhancement is based on the motives of achievement and dominance over others; therefore, it may conflict with Self Transcendence values, which are based on motives such as wanting the well-being of other people and caring about equality [43].

Openness to Change - Conservation: Conservation includes the values of security, conformity and tradition, which are based on the continuity of traditions and the preservation of order. On the other hand, Openness to Change values include self-direction and stimulation values, which include the desire for independent thought and behaviour. Schwartz stated that these value groups would be unlikely to be observed at the same time because their underlying motives would create incompatibility [43].

**Fig. 2:**
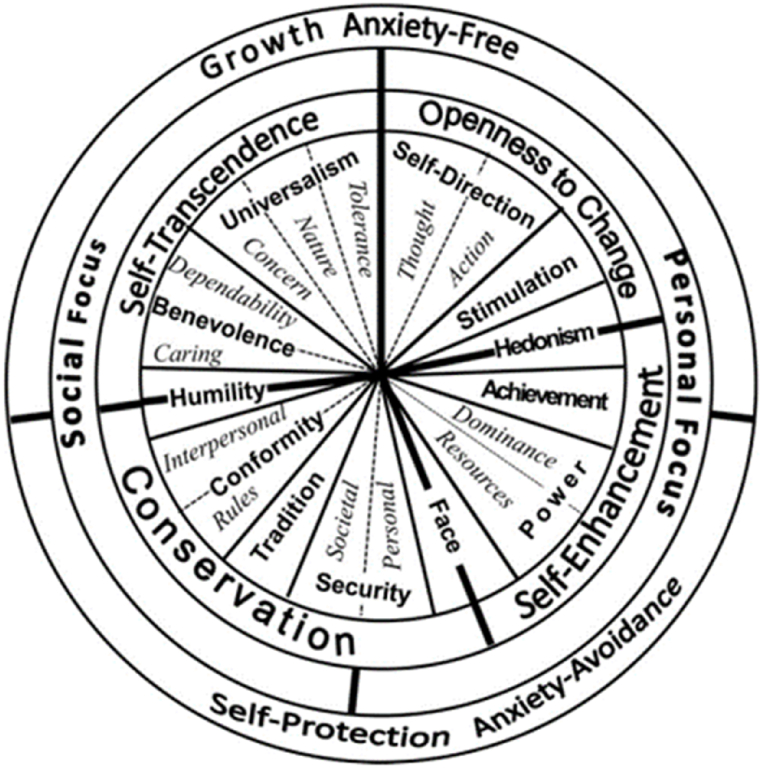
Revised Values and value dimensions [42].

The values and value dimensions study by Schwartz et al. [42] is presented in a circular shape in Fig. 2. This figure shows the three outer layers of circles to explain the conceptual foundations that are effective in ranking values. The top values in the outermost layer refer to *growth-anxiety free* values that motivate individuals in carefree situations. On the other hand, the bottom values in the outermost part of the circle refer to *self-protection* values that aim to protect individuals from anxiety and threat.

*Openness to change value* means readiness for new ideas, behaviours and experiences and is contrasted with the *conservation value*, which is restrictive, values order and avoids change. *Self-enhancement* value means focusing on one's own interests and is contrasted with *self-transcendence* value which means giving up one's own interests for the sake of others [42]. In the theory, hedonism, achievement, face and humility are boundary values. Accordingly, the boundary values were analysed using both upper values. The reasons for being boundary values are clearly stated in the research of Schwartz et al. [42].

### 1.4 Fatalistic tendencies

While the word fatalism does not have a precise meaning, its meaning varies across cultures and religions. In general, however, fatalism can be associated with the tendency to believe that individuals are governed by an invisible force - often referred to as "Destiny" - rather than by their will [44].

There is an overlap between fatalism and superstition, both conceptually and in practice [45]. Superstition is a belief that has no clear cause, emerges socially and affects the behaviour of the person [46]. Most of these ancient beliefs have no scientific basis and have been passed down from generation to generation from family elders or clergy with some misinformation. These beliefs can be found in many societies and cultures [47].

Fatalism has a significant effect on determining a wide range of individual behaviours such as decision-making, occupational choices, health screening behaviours, and natural disaster preparedness. Studies have found that fatalism prevents individuals from adopting self-protection behaviours in problems such as natural disasters [44]. In research on stress and coping, fatalism ("external control") is almost universally cited as a risk factor for poor outcomes, in part because fatalism prevents taking action or seeking help when needed [48].

### 1.5 Safety performance (SP)

SP can be defined as the actions people take in their occupations to ensure their own individual health and safety as well as the health and safety of the environment in which they live [49]. Higher levels of SP are associated with fewer occupational accidents [50].

Griffin and Neal [51] examined SP to differentiate between safe and unsafe behaviours in a safe work environment. In this framework, SP has two dimensions: compliance and participation. Safety compliance includes behaviours such as complying with safety procedures/instructions and conducting work safely, usually referring to mandatory behaviours; while safety participation refers to voluntary behaviours, such as helping co-workers, promoting the safety program in the organization, showing initiative and making efforts to improve safety in the workplace [52].

### 1.6 Safety motivation (SM)

The first research on the effects of SM in determining safety behaviour dates back to the late 1970s [53]. Motivation theories explain why people choose to engage in different safety-related actions and how people's beliefs relate to their safety behaviours [54].

Klein [55] defined human motivation as "a set of psychological processes that cause behaviour to initiate, take direction, intensity and persistence". According to Griffin and Neal [51], SM can be defined as the willingness to exhibit safety behaviours in the workplace. This concept increases employees' awareness, interest and willingness to perform better SP [56]. Christian et al. [57], as a result of their meta-analysis study, stated that SM has a positive relationship with safety behaviours, while experiencing work accidents has a negative relationship.

The aim of SM is to prevent accidents and injuries at the desired level of safety using scientific principles and procedures/instructions. SM influences employees' adherence to safety procedures/instructions and has also been found to ensure the safety of not only employees, but also organizations and even society [58].

### 1.7 Safety risk perception

Risk perception is expressed as the ability of individuals to distinguish a certain amount of risk in the face of natural, technological or social risks and hazards. This ability is mediated through the beliefs, attitudes, judgments and emotions of groups, communities or organizations [59]. Empirical factors influencing unsafe behaviour/risk-taking behaviour are many. Some studies have been conducted in developing countries to find the relationship between employee safety conscious behaviour, risk perception and national cultures. For example, one study found that the safety responsibilities of local construction site workers in Pakistan were related to their attitudes and perceived risks, and that this relationship was explained by the national culture of the country [60].

Risk perception and safety culture are similar in their assessments of beliefs, attitudes, social practices and social values. However, the concept of safety culture usually refers to the "work environment", while risk perception has no such limitation [61].

Recent research has shown that in Asian developing countries, health and safety professionals, employees, and local cultures are influenced by beliefs and religious factors that may lead employees to engage in unsafe or risky behaviours [59]. The aim of this study is to develop a model to understand human behaviour, which is one of the most important causes of occupational accidents, and to develop a model to examine the effect of safety culture, which is a component of culture that forms the basis of human behaviour, on behaviour and to determine the values of employees that form the basis of safety culture and to examine the effects of these values on fatalistic tendencies, risk perception, SM and SP. In short, individual factors constitute the motivation for this study.

## 2 Materials and method

In this section of the study, the research questions, research assumptions, research model, population and sample of the study, data collection tools and analysis are discussed.

### 2.1 Research model and research hypotheses

**Fig. 3:**
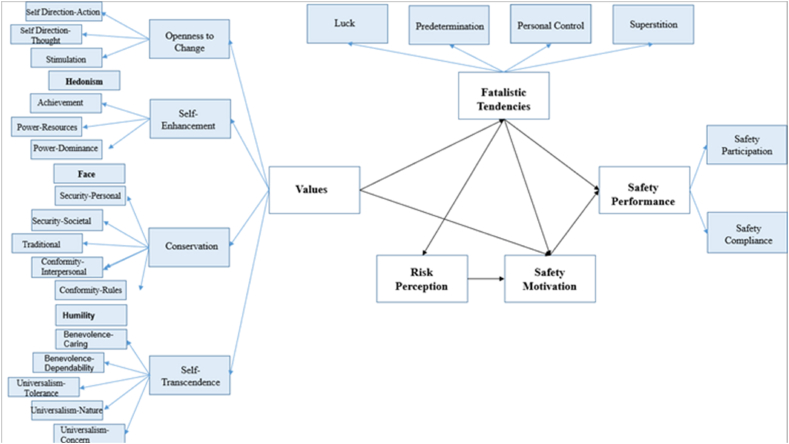
Model of the study.

The conceptual model created according to the assumptions of the model to be used within the scope of the research is shown in Fig. 3. According to Fig. 3, the values latent variable consists of 4 dimensions; openness to change, self-enhancement, self-transcendence and conservation. There are a total of 19 basic values (sub-dimensions) connected to these 4 dimensions. The effects of these 19 basic values on fatalistic tendencies, SM, risk perception and their effects on SP will be examined.

Hypotheses:

• H1. Values sub-dimensions affect fatalistic tendencies.
• H2. Values sub-dimensions affect SM.
• H3. Values sub-dimensions affect risk perception.
• H4. Values sub-dimensions affect behaviour.
• H5. Fatalistic tendencies are the mediating variable between values and behaviour.
• H6. SM is a mediating variable between values and behaviour.
• H7. Risk perception is a mediating variable between values and behaviour.

### 2.2 Research sample

General sample size recommendations used in path analysis and SEM studies are mainly based on the number of observable variables in the variance-covariance matrix or the number of data points. Gorsuch [62], stated that there should be at least 5 participants for each variable and at least 200 participants in total. Tabachnick & Fidell [63] stated that there should be *8 + 50 participants per variable. Another sample size calculation is the absolute sample size. Accordingly, Comrey & Lee [64] stated that a sample size of at least 200 is reasonable, but a sample size of more than 500 is very good. Considering the different sample size calculations on the subject, a sample size of at least 701 people was found within the scope of this research.

**Fig. 4:**
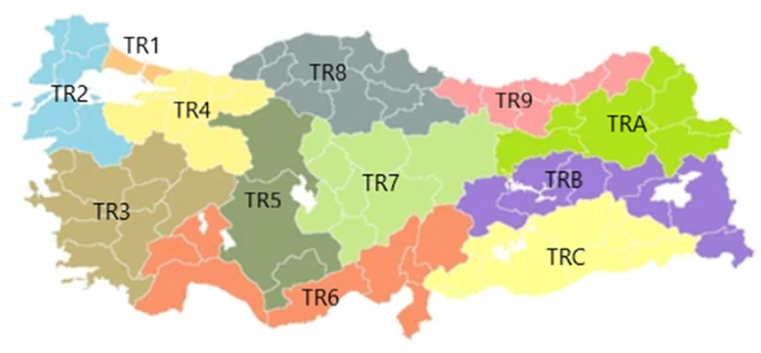
Nuts regions.

In line with Türkiye's harmonization process with the European Union, NUTS-12 regions were established in accordance with the Law No. 2002/4720 issued by the Council of Ministers, taking into account economic indicators, population, regional development plans, social and geographical similarities. This regional distinction has become a frequently used variable in analysing the demographic, social, cultural and economic differences between regions of the country. For this reason, in this study, data were collected over the NUTS-12 region. Fig. 4 shows the NUTS-12 region. Accordingly; TR1; Istanbul, TR2; West Marmara, TR3; Aegean, TR4; East Marmara, TR5; West Anatolia, TR6; Mediterranean, TR7; Middle Anatolia, TR8; West Black Sea, TR9; East Black Sea, TRA; Northeast Anatolia, TRB; Middle East Anatolia, TRC; Southeast Anatolia.

### 2.3 Measurement tools for quantitative research

a) **Demographic Information Form:** There are questions to obtain general socio-demographic information of the participants in the study.
b) **Portrait Values Questionnaire-57:** The form of the 'Portrait Values Questionnaire-57′ prepared in English by Schwartz et al. [42] and translated adapted into Turkish by Kürşad Demirutku and Özlem Dirilen-Gümüş, who were also in the Türkiye group of the same study, was used in the study. It consists of 57 items.
c) **Fatalistic tendencies:** It was developed by Kaya & Bozkur [65]. The scale consists of four sub-dimensions, namely Predetermination, Personal Control, Superstition and Luck, and 24 items in total. All of the sub-dimensions are external control-oriented and attribute control to factors outside the self. Since personal control is reverse coded, it will be expressed as lack of personal control.
d) **Risk Perception:** The scale developed by Olcay [66] consists of 3 items.
e) **Safety Motivation:** The determinants of SP are the SM scale that Neal et al. [52]. The Turkish adaptation and validity study of the scale was conducted by Sakallı et al. [67]. It consists of 11 items.
f) **Safety Performance(Behaviour):** SP components consist of the behaviour scale made by Neal et al. [52]. The Turkish adaptation and validity study of the scale was conducted by Sakallı et al. [67]. The scale consists of two sub-dimensions: safety compliance and safety participation. It consists of 8 items.

Ethical approval certificate dated December 21, 2021 and numbered 2021/72 was obtained from Istanbul Medeniyet University for the research.

The fit indices to be used in the research are given in Table 2. In this table, the fit values and acceptable values of the indices and the properties of the related index are given.

**Table 2:** Fit Indices to be used in the study.

Assessment Indicator	Fit indices	Description	Value Ranges
Inferential fit index	χ^2^/sd	Chi-square degrees of freedom should be used when N > 200.	2 < χ^2^/sd ≤ 3: Acceptable fit 0 ≤ χ2/sd ≤ 2: Good fit
Alternative fit index	RMSEA	The most popular of the fit indices is RMSEA.	0.05 ≤ RMSEA ≤0.08:Acceptable fit 0.00 ≤ RMSEA ≤0.05: Good fit
	SRMR	It is the standardized difference between the observed and estimated covariances.	0.05<SRMR≤0.10: Acceptable fit 0<SRMR≤0.05: Good fit
Incremental fit index	CFI	It is one of the most widely used indices. It assumes no relationship between the measures, with the logic of comparing the proposed model with the null model.	0.90 ≤ CFI≤0.95: Acceptable fit 0.95 < CFI≤1.00: Good fit
Descriptive Fit Index	NNFI(TLI)	It tries to correct for negative bias by including the null model and the researcher's model and degrees of freedom.	0.90 ≤ NNFI (TLI) < 0.95: Acceptable fit 0.95 ≤ NNFI (TLI) ≤ 1.00: Good fit
	AGFI	GFI is the fit index adjusted for sample size and this index is used when the sample is large.	0.85 ≤ AGFI <0.90: Acceptable fit 0.90 ≤ AGFI ≤1.00: Good fit

*Source:* [[68], [69], [70], [71], [72], [73]].

### 2.4 Data analysis

In line with the purpose of the study, path analysis was used to examine the relationship between individuals' value dimensions, fatalistic tendencies, SM, SP and risk perception behaviour. The purpose of path analysis is to determine the model that best fits the available data.

Parametric analysis techniques (independent samples *t*-test, one-way ANOVA were used to compare the participants' scores on the relevant scales with variables such as gender, geographical region, age and educational status. The necessary assumptions were checked before using parametric analysis techniques. The normality assumption was examined by calculating the skewness and kurtosis coefficients of the data. The fact that the skewness and kurtosis coefficients are within the range of ±2 is considered sufficient to accept the assumption of normal distribution [74]. In this direction, when the total of the scales and the dimensions of the scales were examined, it was determined that all of them accepted the assumption of normal distribution. For the model assumption of the research, AMOS was used for path analysis and SPSS 24.0 was used for other analyses. The analysis results were analysed at 95 % confidence level.

## 3 Findings

### 3.1 Findings regarding variables

Table 3 provides demographic information about the 701 respondents. Accordingly, data were collected from NUTS-12 regions in proportion to the population of Türkiye and demographic information deemed important for the research was collected.

**Table 3:** Demographic information of the study.

Variables	Groups	Frequency (N = 701)	%
**Gender**	Woman	336	47.9
	Male	365	52.1
**Occupation**	Health workers	38	5.4
	Unskilled workers	323	46.1
	Engineer/Architect	51	7.3
	Educator	94	13.4
	Administrator	66	9.4
	Others	129	18.4
**Marital Status**	Married	500	71.3
	Single	201	28.7
**Education Status**	High school and below	172	24.5
	Associate degree	78	11.1
	Bachelor's degree	410	58.5
	Post-graduate	41	5.8
**Age**	29 years and below	256	36.5
	30–39 years old	268	38.2
	40 years and older	177	25.2
**Accident Status**	Had an accident	299	42.7
	had not an accident	402	57.3
**NUTS-12 Region**	TR1	130	18.5
	TR2	31	4.4
	TR3	88	12.6
	TR4	72	10.3
	TR5	70	10.0
	TR6	88	12.6
	TR7	33	4.7
	TR8	40	5.7
	TR9	21	3.0
	TRA	20	2.9
	TRB	33	4.7
	TRC	75	10.7
**Monthly Income**	7500 TL and below	274	39.1
	Between 7.501 and 9.000 TL	235	33.5
	9.001 TL and above	192	27.4
**Child Status**	Yes	466	66.5
	No	235	33.5
**Work life**	Up to 5 years	357	51.0
	6–10 years	179	25.5
	11 years and above	165	23.5

#### 3.1.1 Examining the relationship of values classified in terms of anxiety with fatalistic tendencies, safety motivation, risk perception and safety performance by path analysis

Considering the goodness of fit of the model (x^2/sd^: 2.5 acceptable fit; SRMR: 0.02 good fit; AGFI: 0.98 good fit; CFI: 0.99 good fit; RMSEA: 0.04 good fit; TLI: 0.99 good fit), it was determined that the research model was in good fit (p values were all p < .01).

Researchers in behavioural and cognitive sciences have been advised to report and interpret effect sizes in their research papers [75]. In standardized regression coefficients (β), coefficients around 0.10 are considered as small effects, around 0.30 as medium effects and coefficients above 0.50 as large effects. However, there are no sharp boundaries between these coefficients. For example, there are no sharp boundaries between 0.49 and 0.50 [76].

**Fig. 5:**
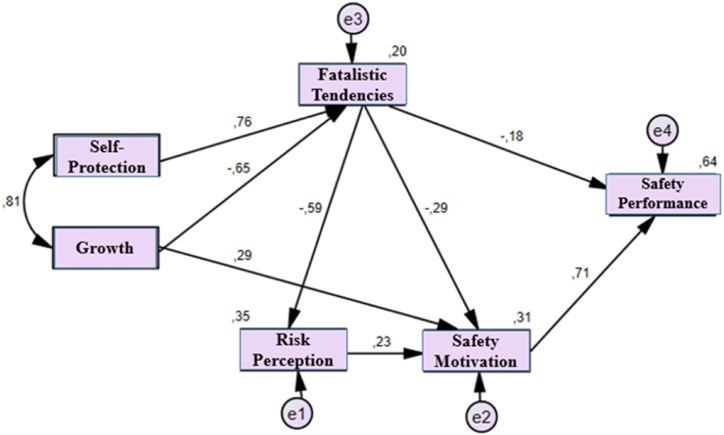
Research Model of Values in terms of Anxiety.

Fig. 5 shows that the β between Growth-anxiety free values and fatalistic tendencies is negative −0.65, and the β between self-protection-anxiety avoidance values and fatalistic tendencies is 0.76. Accordingly, both β indicate large effects. The β between Growth- Anxiety free values and SM is 0.29, which is close to medium-sized effect. The β and the magnitude of the effect between fatalistic tendencies and risk perception, SM and SP were found to have a large effect with −0.59, a medium effect with −0.29 and a small effect with −0.18, respectively. In addition, β between risk perception and SM was found to be 0.23 and finally, β between SM and SP was observed to have a large effect with 0.71.

20 % of the total change in fatalistic tendencies is explained by Growth - anxiety free values and Self-protection - Anxiety avoidance values. 35 % of the total change in risk perception is explained by fatalistic tendencies. Similarly, 35 % of the total change in SM is explained by fatalistic tendencies and risk perception. Finally, 64 % of the total change in SP is explained by the model.

Table 4 shows the total effect of indirect and direct effects. Accordingly, the effect between Growth-anxiety free values and SP is a large effect with 0.52; the effect between Self-Protection-Anxiety Avoidance values and SP is a medium effect with −0.36; the effect between Fatalism tendencies and SP is a large effect with −0.48; the effect between risk perception and SP is a small effect with 0.16; and finally, the effect between SM and SP is a large effect with 0.71. This means that when growth-anxiety free values increases by 1 standard deviation, SP increases by 0.52 standard deviations, while when Self Protection - avoidance values increases by 1 standard deviation, SP decreases by 0.36 standard deviations.

**Table 4:** Standardized total effect of path analysis.

	Growth – anxiety free	Self-Protection - anxiety avoidance	Fatalistic Tendencies	Risk Perception	Safety Motivation
**Fatalistic Tendencies**	-,65	,76	,00	,00	,00
**Risk Perception**	,39	-,45	-,59	,00	,00
**Safety Motivation**	,57	-,32	-,42	,23	,00
**Safety Performance**	,52	-,36	-,48	,16	,71

As a result, all hypotheses were accepted and other findings examined the hypotheses in depth. (H1. Values sub-dimensions affect fatalistic tendencies; H2. Values sub-dimensions affect SM; H3.Values sub-dimensions affect risk perception; H4. Values sub-dimensions affect behaviour; H5. Fatalistic tendencies are the mediating variable between values and behaviour; H6. SM is a mediating variable between values and behaviour; H7. Risk perception is a mediating variable between values and behaviour.)

#### 3.1.2 Examining the relationship between values classified in terms of the four top values and fatalistic tendencies, safety motivation, risk perception and safety performance with path analysis

**Fig. 6:**
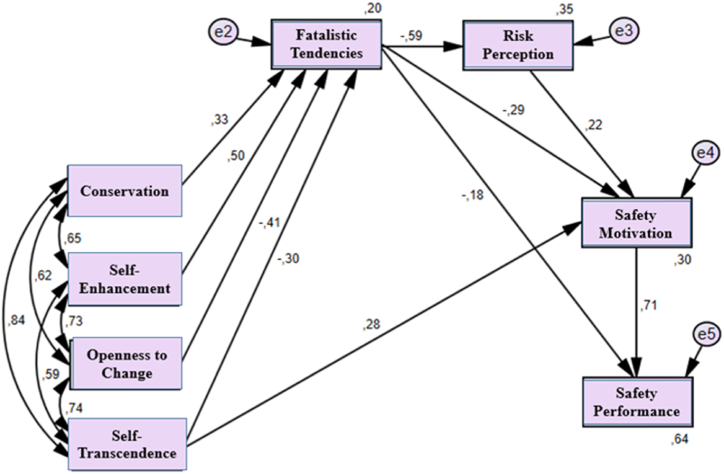
Research Model of Values in terms of the Four Superordinate Values.

Looking at the goodness of fit of the model shown in Fig. 6 (x2/sd: 2.02 acceptable fit; SRMR: 0.02 good fit; AGFI: 0.98 good fit; CFI: 0.99 good fit; RMSEA: 0.04 good fit; TLI: 0.99 good fit), it has been determined that the research model is in good fit (p values are all p < .01).

According to Fig. 6, conservation value, self-enhancement value, openness to change value and self-transcendence value were found to affect fatalistic tendencies and the standardized regression coefficients and magnitudes were found to have a medium effect with 0.33, a large effect with 0.50, a medium effect with −0.41, and a medium effect with −0.30, respectively. Conservation and self-enhancement values positively affect fatalistic tendencies, while openness to change and self-transcendence values negatively affect fatalistic tendencies. In addition, self-transcendence value was found to affect SM and β values were found to be medium in magnitude with 0.28. The β values and magnitudes between fatalistic tendencies and risk perception, SM and SP were observed to have a large effect with −0.59, a medium effect with −0.29 and a small effect with −0.18, respectively. The β value between risk perception and SM was found to be 0.22, and the β values between SM and SP were found to be a large effect with 0.71. Hypotheses H1, H2, H3, H4, H5, H6, H7 and H8 of the research are accepted.

Table 5 shows the total effect of direct and indirect effects. Accordingly, the effect between conservation and SP is small with -,16; the effect between self-enhancement and SP is medium with -,24; the effect between self-transcendence and SP is medium with,34; the effect between openness to change and SP is small with,20. Similarly, it was found that the effect between risk perception and SP was a small effect with,16, the effect between fatalistic tendencies and SP was a large effect with -,48, and finally the effect between SM and SP was a large effect with,71.

**Table 5:** Standardized total effect of values for the four upper values.

	Conservation	Self Enhancement	Self Transcendence	Openness to Change	Fatalistic Tendencies	Risk Perception	Safety Motivation
**Fatalistic Tendencies**	0.33	0.50	−0.30	−0.41	0.00	0.00	0.00
**Risk Perception**	−0.19	−0.30	0.18	0.24	−0.59	0.00	0.00
**Safety Motivation**	−0.14	−0.21	0.41	0.17	−0.42	0.22	0.00
**Safety Performance**	−0.16	−0.24	0.34	0.20	−0.48	0.16	0.71

#### 3.1.3 Partial correlation of values categorized in terms of anxiety with fatalistic tendencies, safety motivation, risk perception and safety performance

According to the meta-analysis results of 708 studies, it has been stated that the correlation coefficient (r) between 0.10 and 0.19 has a small effect, between 0.20 and 0.29 has a medium effect, and 0.30 and above has a large effect [77]. According to Table 6, there is a medium (,27) correlation between Growth values and SM, a medium (0,24) correlation between SP, a large (-,41) correlation between fatalistic tendencies, a large (,30) correlation between risk perception and a large (-,90) correlation between self-protection values. There is a medium (,27) correlation between Self-Protection values and SM, a medium (0,25) correlation between SP, a large (-,45) correlation between fatalistic tendencies, and a large (,33) correlation between risk perception. There is also a large (0.78) correlation between SP and SM, a large (−0.52) correlation between fatalistic tendencies, and a large (0.43) correlation between risk perception.

**Table 6:** Partial Correlation Table of Values Classified in terms of Anxiety with Fatalistic Tendencies, Safety Motivation, Risk Perception and Safety Performance.

Correlation									
Control Value			Safety Motivation	Safety Performance	Fatalistic Tendencies	Risk Perception	Growth - anxiety free	Self-Protection - anxiety avoidance	
**Average of values**	**Safety Motivation**	Correlation	1,00						
		Sig.(2-tailed)	.						
	**Safety Performance**	Correlation	,775	1,00					
		Sig.(2-tailed)	,000	.					
	**Fatalistic Tendencies**	Correlation	-,472	-,516	1,00				
		Sig.(2-tailed)	,000	,000	.	,			
	**Risk Perception**	Correlation	,435	,432	-,588	1,00			
		Sig.(2-tailed)	,000	,000	,000	.			
	**Growth - anxiety free**	Correlation	,270	,240	-,407	,303	1,00		
		Sig.(2-tailed)	,000	,000	,000	,000	.		
	**Self-Protection - anxiety avoidance**	Correlation	-,271	-,246	,446	-,333	-,904	1,00	
		Sig. (2-tailed)	,000	,000	,000	,000	,000	.	

##### 3.1.3.1 Partial correlation of value dimensions with fatalistic tendencies, safety motivation, risk perception and safety performance

According to Table 7, the correlations between the 19 value dimensions and SM, SP, risk perception and fatalistic tendencies were examined. Only those that differed significantly with all these four scales and the significant difference was greater than 0.1 were included in the table.

**Table 7:** 19 partial correlation of value dimensions with fatalistic tendencies, safety motivation, risk perception and safety performance.

Control Value			Safety Motivation	Safety Performance	Risk Perception	Fatalistic Tendencies
**Average of values**	**Power - Resources**	Correlation	−0.202	−0.210	−0.252	0.335
		Sig.(2-tailed)	0.000	0.000	0.000	0.000
	**Power - Dominance**	Correlation	−0.215	−0.214	−0.313	0.272
		Sig.(2-tailed)	0.000	0.000	0.000	0.000
	**Self Direction -Action**	Correlation	0.212	0.227	0.259	−0.351
		Sig.(2-tailed)	0.000	0.000	0.000	0.000
	**Self Direction - Thought**	Correlation	0.196	0.167	0.213	−0.325
		Sig.(2-tailed)	0.000	0.000	0.000	0.000
	**Universalism - Nature**	Correlation	0.146	0.164	0.136	−0.179
		Sig.(2-tailed)	0.000	0.000	0.000	0.000
	**Universalism - Concern**	Correlation	0.183	0.224	0.224	−0.297
		Sig.(2-tailed)	0.000	0.000	0.000	0.000
	**Tradition**	Correlation	−0.223	−0.224	−0.178	0.292
		Sig.(2-tailed)	0.000	0.000	0.000	0.000
	**Benevolence - Dependability**	Correlation	0.127	0.113	0.228	−0.224
		Sig.(2-tailed)	0.001	0.003	0.000	0.000
	**Benevolence - Caring**	Correlation	0.143	0.096	0.173	−0.288
		Sig.(2-tailed)	0.000	0.011	0.000	0.000
	**Conformity - Interpersonal**	Correlation	0.131	0.141	0.131	−0.261
		Sig.(2-tailed)	0.001	0.000	0.000	0.000
	**Conformity - Rules**	Correlation	−0.110	−0.084	−0.142	0.174
		Sig.(2-tailed)	0.003	0.026	0.000	0.000
	**Security - Personal**	Correlation	0.172	0.182	0.151	−0.209
		Sig.(2-tailed)	0.000	0.000	0.000	0.000

According to the table, Power (Resources and Dominance), Tradition, Conformity-Rules values are negatively correlated with SM, risk perception and SP, while they are positively correlated with fatalistic tendencies. Security-personal, Benevolence (Caring and Dependability), Conformity-interpersonal, Universalism (Nature and Concern), Self-direction (Thought and Action) values are positively correlated with SM, risk perception and SP, while they are negatively correlated with fatalistic tendencies.

#### 3.1.4 Changes in fatalistic tendencies, safety motivation, safety performance, risk perception and value structures by accident status

When Table 8 is examined, it is seen that the averages of SM and SP do not show a significant difference according to the accident status (p > 0.05). However, it was found that people who had an work accident had higher fatalistic tendencies and lower risk perception than those who did not have an work accident. Only the values with significant differences related to accidents are presented in the table (p < 0.05). Accordingly, it was found that people who had an work accident were more likely to have power-dominance, power-resources, conformity-rules and tradition values, which are directly related to SP, SM, risk perception and fatalistic tendencies among the 19 basic values and negatively affect SP, compared to the group who did not have an work accident (p < 0.05).

**Table 8:** Table of changes in fatalistic tendencies, safety performance, safety performance, risk perception by accident status.

Variables		Accident Status	N	$\bar{X}$	Ss	p	Differences between groups
**Safety Motivation**	1.	Had an accident	299	4.02	0.61	0**.52**	–
	2.	Had not an accident	402	4.05	0.61		
**Safety Performance**	1. 2.	Had an accident	299	3.97	0.64	0**.51**	–
		Had not an accident	402	4.01	0.65		
**Fatalistic Tendencies**	1. 2.	Had an accident	299	2.89	0.60	0**.00**	1 > 2
		Had not an accident	402	2.71	0.64		
**Risk Perception**	1. 2.	Had an accident	299 402	2.82	0.86 0.92	0**.00**	2 > 1
		Had not an accident		3.05			
**Conservation**^a^	1.	Had an accident	299	4.22	0.50	0**.01**	1 > 2
	2.	Had not an accident	402	4.11	0.51		
**Self Enhancement**^a^	1. 2.	Had an accident	299	4.00	0.56	0**.00**	1 > 2
		Had not an accident	402	3.88	0.55		
**Self-Protection - Anxiety Avoidance**^a^	1. 2.	Had an accident	299	4.11	0.48	0**.00**	1 > 2
		Had not an accident	402	3.97	0.48		
**Tradition**	1. 2.	Had an accident	299	4.11	0.81	0**.00**	1 > 2
		Had not an accident	402	3.79	1.02		
**Conformity- Rules**	1. 2.	Had an accident	299	4.11	0.64	0**.00**	1 > 2
		Had not an accident	402	3.92	0.78		
**Power - Resources**	1. 2.	Had an accident	299	3.58	0.93	0**.00**	1 > 2
		Had not an accident	402	3.35	0.93		
**Power- Dominance**	1. 2.	Had an accident	299	3.64	0.85	0**.00**	1 > 2
		Had not an accident	402	3.37	0.81		

a Upper Value Classifications.

Similarly, it was found that people who had experienced a work accident were more likely to have conservation, self-enhancement, self-protection values, which are directly related to SP, SM, risk perception and fatalism tendencies and negatively affect safety behaviours, than the group who had not experienced a work accident (p < 0.05).

#### 3.1.5 Changes in fatalistic tendencies, safety motivation, safety performance, risk perception and value structures by educational background

According to Table 9, only the findings that differed significantly were included (p < 0.05). Accordingly, it was found that the power-resources value was higher in high school and below, associate and bachelor's degree graduates compared to postgraduate graduates; the power-dominance value was higher in high school and below and bachelor's degree graduates compared to postgraduate graduates. It was found that the self-direction-action value was higher in postgraduate graduates than in high school and below, associate degree and bachelor's degree graduates, and similarly, bachelor's degree graduates were higher than high school and below graduates. Self-direction-thought value was higher for postgraduate graduates than high school and below and associate degree graduates, and higher for bachelor's degree graduates than high school and below graduates. It is seen that the value of universalism-nature is higher for individuals with a bachelor's degree than individuals with a high school diploma and below. The value of tradition was found to be higher for high school and below and bachelor's degree graduates than postgraduate degree. It was found that the value of growth-anxiety free was higher for individuals with bachelor's and postgraduate degrees than for individuals with associate's and below bachelor's degrees, and the value of self-protection- anxiety avoidance was higher for individuals with bachelor's degrees than for individuals with postgraduate degrees. The openness to change value was found to be higher in individuals with bachelor's and postgraduate degrees than in individuals with high school and below, and the self-enhancement value was higher in individuals with bachelor's degrees than in individuals with postgraduate degrees. When the fatalistic tendencies are examined, it has been determined that individuals with high school and below degrees are higher than those with associate, postgraduate and bachelor's degrees, however, individuals with associate and bachelor's degrees have higher fatalistic tendencies than individuals with postgraduate degrees. It is seen that risk perception is higher for postgraduate degree than high school and below, associate degree and bachelor's degree. Finally, it was determined that the SM of postgraduate graduates is higher than high school and below degrees.

**Table 9:** Changes in fatalistic tendencies, safety motivation, safety performance, risk perception and value structures by educational status.

Variables		Educational Status	N	$\bar{X}$	Ss	p	Differences between groups
**Power-Resources**	1.	High school and below	172	3.52	0.86	0**.00**	1 > 4, 2 > 4, 3 > 4
	2.	Associate degree	78	3.42	0.83		
	3.	Bachelor's degree	410	3.48	0.97		
	4.	Post-graduate	41	2.94	0.93		
**Power-Dominance**	1.	High school and below	172	3.50	0.79	0**.00**	1 > 4, 3 > 4
	2.	Associate degree	78	3.30	0.76		
	3.	Bachelor's degree	410	3.56	0.85		
	4.	Post-graduate	41	3.00	0.85		
**Self Direction- Action**	1.	High school and below	172	4.23	0.72	0**.00**	3 > 1,
	2.	Associate degree	78	4.31	0.62		4 > 1,
	3.	Bachelor's degree	410	4.41	0.56		4 > 2,
	4.	Post-graduate	41	4.71	0.46		4 > 3
**Self Direction- Thought**	1.	High school and below	172	4.16	0.68	0**.00**	3 > 1, 4 > 1, 4 > 2
	2.	Associate degree	78	4.24	0.60		
	3.	Bachelor's degree	410	4.37	0.58		
	4.	Post-graduate	41	4.63	0.40		
**Universalism- Nature**	1.	High school and below	172	4.18	0.77	0**.00**	3 > 1
	2.	Associate degree	78	4.22	0.69		
	3.	Bachelor's degree	410	4.42	0.60		
	4.	Post-graduate	41	4.38	0.59		
**Humility**	1.	High school and below	172	3.87	0.76	0**.01**	3 > 4
	2.	Associate degree	78	3.86	0.68		
	3.	Bachelor's degree	410	3.95	0.73		
	4.	Post-graduate	41	3.57	0.81		
**Tradition**	1.	High school and below	172	4.02	0.89	0**.00**	1 > 4, 3 > 4
	2.	Associate degree	78	3.83	0.90		
	3.	Bachelor's degree	410	3.95	0.96		
	4.	Post-graduate	41	3.46	1.01		
**Growth-Anxiety free**^a^	1.	High school and below	172	4.16	0.76	0**.01**	3 > 1, 4 > 1
	2.	Associate degree	78	4.22	0.68		
	3.	Bachelor's degree	410	4.30	0.73		
	4.	Post-graduate	41	4.33	0.81		
**Self Protection - Anxiety avoidance**^a^	1.	High school and below	172	4.02	0.50	0**.00**	3 > 4,
	2.	Associate degree	78	3.93	0.43		
	3.	Bachelor's degree	410	4.07	0.49		
	4.	Post-graduate	41	3.82	0.40		
**Openness to Change**^a^	1.	High school and below	172	4.10	0.63	0**.00**	3 > 1, 4 > 1
	2.	Associate degree	78	4.20	0.54		
	3.	Bachelor's degree	410	4.28	0.50		
	4.	Post-graduate	41	4.37	0.39		
**Self Enhancement**^a^	1.	High school and below	172	3.91	0.57	0**.01**	3 > 4
	2.	Associate degree	78	3.86	0.47		
	3.	Bachelor's degree	410	3.98	0.57		
	4.	Post-graduate	41	3.71	0.45		
**Fatalistic Tendencies**	1.	High school and below	172	2.95	0.58	0**.00**	1 > 2, 1 > 3, 1 > 4, 2 > 4, 3 > 4
	2.	Associate degree	78	2.70	0.67		
	3.	Bachelor's degree	410	2.78	0.62		
	4.	Post-graduate	41	2.29	0.57		
**Risk Perception**	1.	High school and below	172	2.81	0.90	0**.00**	4 > 1, 4 > 2, 4 > 3
	2.	Associate degree	78	2.98	1.00		
	3.	Bachelor's degree	410	2.94	0.87		
	4.	Post-graduate	41	3.59	0.73		
**Safety Motivation**	1.	High school and below	172	3.96	0.60	0**.01**	4 > 1, 4 > 3
	2.	Associate degree	78	4.08	0.56		
	3.	Bachelor's degree	410	4.03	0.62		
	4.	Post-graduate	41	4.30	0.55		

a Upper Value Classifications.

#### 3.1.6 Türkiye fatalistic tendencies map

**Fig. 7:**
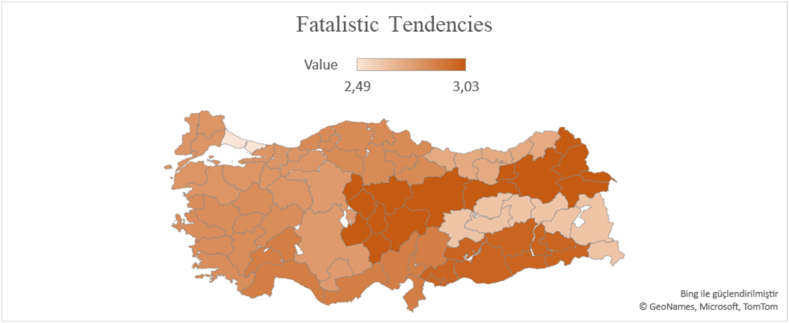
Türkiye NUTS-12 region fatalistic tendencies map.

According to Fig. 7, the region with the lowest fatalistic tendencies is TR1 (Istanbul) with an average of 2.49. Istanbul is followed by TRB region with an average of 2.62. TR9 (2.72), TR5 (2.78), TR4 (2.8), TR2 (2.8), TR3 (2.83) come next. The regions with the most fatalistic tendencies are TR7 (3.03) and TRA (3.03). These regions are followed by TRC (2.99) and TR6 (2.89).

#### 3.1.7 Value dimensions with the highest and Lowest Mean in Turk Society

**Fig. 8:**
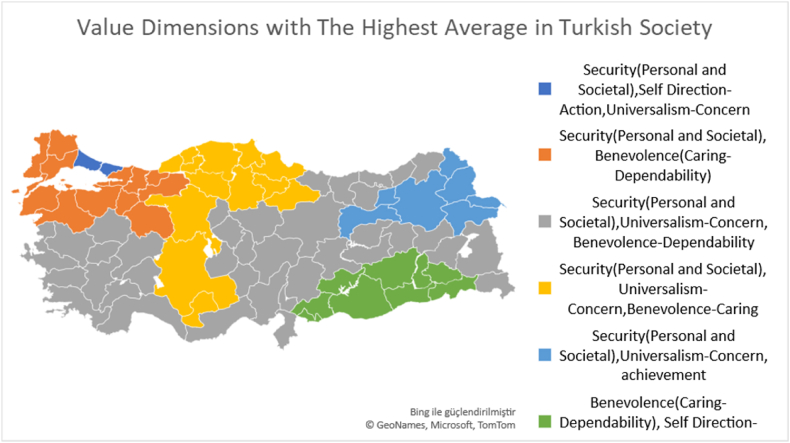
Four value dimensions with the highest average in Turkish society.

Fig. 8 shows the 4 value dimensions with the highest average in Turk society. Accordingly, it is seen that security- (personal-societal) values are prominent in all 11 regions except TRC region. In addition, in TR1 region, where fatalistic tendencies are the lowest, self-direction-action and universalism-concern values are also found to be high. In general, it is noteworthy that in addition to security (personal-societal) values, universalism-concern value is also present in almost every region. It is seen that the value dimension with the highest average in TR3, TR5, TR6, TR7, TR9 and TRB region is the same.

**Fig. 9:**
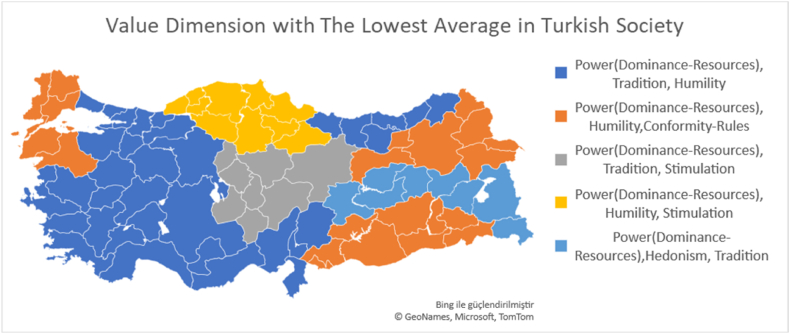
Four value dimensions with the lowest mean in turk society.

Fig. 9 shows the 4 value dimensions with the lowest average in the Turk society. Accordingly, it is seen that the 4 value dimensions with the lowest average in TR1, TR3, TR4, TR5, TR6 and TR9 regions are the same. These values are power-resources, power-dominance, tradition and humility. Similarly, power-dominance and power-resources are among the values with the lowest mean in each region. When looking at the values with the lowest mean, it was found that the value of conformity-rules was found in TRA and TRC regions, which showed the highest fatalistic tendencies together with TR2.

## 4 Discussion

Since the *self-enhancement* value is based on the motives of success and dominance over others and will shape their behaviours within their own ambitions [42], it is thought to affect SP, SM and risk perception negatively and fatalism tendencies positively. Thus, it is thought that *self transcendence* values based on motives such as wanting the well-being and welfare of other people and giving importance to equality [42] positively affect SM, SP and risk perception and negatively affect fatalistic tendencies.

In a similar study, Mearns & Yule [78] stated that the only cultural variables that significantly predict risk taking are masculinity and power distance among Hofstede's cultural value dimensions. They said that employees with higher scores on these variables are more likely to take risks at work. For example, 'Femininity' is about valuing people and relationships, and this can extend to concern for the health, safety and well-being of others. In contrast, Masculinity, success, advancement and monetary gain were the main motivations for behaviour, not concern for other people. In this study, power-dominance and power-resources values were found to negatively affect SP, SM and risk perception, and positively affect fatalistic tendencies. As a matter of fact, benevolence- (caring, dependability), universalism- (nature, concern) values were found to positively affect SP, SM, and risk perception and negatively affect fatalistic tendencies.

*Conservation value* includes the values of security, conformity and tradition, which are based on the continuity of customs, preservation of order and self-limitation [42]. [79]In Türkiye, the Occupational Health and Safety(OHS) Law was enacted in 2012 and entered into working life [80]. Therefore, it is thought that the OHS phenomenon, which is a concept that cares about the continuation of the past and is not present in past working lives, has a negative effect on SP, SM and risk perception of individuals with high conservatism value, and a positive effect on fatalistic tendencies. *Openness to Change values* include the desire for independent thought and behaviour, self-direction including being open to change and stimulation values [43]. With OHS, which is a new concept in Turk society, it is thought that the active participation of employees in OHS, the importance of their ideas and the introduction of new working principles positively affect the SM, SP and risk perception of individuals who attach importance to the value of openness to change, while thinking that they are not in control, such as fatalistic tendencies, has a negative effect. Because openness to change values include the freedom in people's behaviours, it is an expected result that their behaviours are less likely to be explained by a power other than themselves.

Since the motives underlying conservation and openness to change, self-transcendence and self-enhancement are opposite to each other [43], the results show a consistency within themselves.

*Self protection-anxiety avoidance value*, which serves to cope with anxiety due to uncertainty in the social and physical world, no development, to avoid conflict with people (conformity) and to preserve the existing order (tradition, security) or to actively control the threat (power) [42], in short, to preserve the existing order, is thought to negatively affect SM, SP and risk perception, but positively affect fatalistic tendencies. The opposite value, *growth-anxiety-free values* (hedonism, stimulation, self-direction, universalism, benevolence) represent carefree willingness. These are growth or self-expanding values. It is thought that this is the reason why SP, SM and risk perception are positively affected and fatalistic tendencies are negatively affected in the OHS concept, which is a new concept in the working life of Turk society, in people who adopt values without development-anxiety. Basing values on interests and concerns can help to predict and understand the relationship between values and various attitudes and behaviours (Schwartz, 2012).

A study of 15,454 pilots from 36 airlines operating in 23 countries using the Hoftstede values questionnaire found evidence of national differences among pilots, particularly in the areas of command, attitudes towards automation and attitudes towards rules and procedures. It was stated that pilots' behaviour is determined by one or more of these dimensions, Individualism, Power Distance and Uncertainty Avoidance. For example, pilots with high Power Distance may be more likely to follow orders and comply with standard operating procedures [81,82]. In similar studies, Okolie & Okoye [83] found that aspects of Hofstede's cultural dimensions are related and supportive of hazardous behaviours, risk perception and attitudes towards safety of workers on construction sites. Kheni [84] stated that there is a cultural link between employees' OHS-related behaviours and occupational accidents. In addition, Kheni [84], in his research conducted in Ghana, found that employees used some culture-based words and phrases such as "I adapt to traditions", "fate", "mystery".

Among the self-protection-anxiety avoidance values, only conformity-interpersonal and security-personal values have opposite effects with the other values in this group. While the SM, SP and risk perception of people with high conformity-rules value, which advocates the behaviour of obeying rules, laws and legal obligations, decrease, their fatalistic tendencies increase. The value of obeying-rules was found to be the least common value in Turk society. In this society where the understanding of "rules are meant to be broken" is sometimes adopted, doing something just because it is a rule probably results in negative behaviour. In fact, this is explained in Trompenaars & Hampden-Turner [85] culture study as universalism and particularism. In universalism, it means that the rules are valid for everyone, but in particularism, it means that the rules can be flexed and exceeded according to individuals and groups. They argue that in societies dominated by particularism, the conditions that arise in certain situations are more important than rules. In these societies, close ties with family or the environment are of great importance, and the bonds formed by relationships are stronger than rules. Therefore, behaviours in these communities can be done in order to maintain relationships and avoid being excluded from the community [86]. Accordingly, in this study, since social values are more important in Turk society and since the *conformity-interpersonal value*, which advocates avoiding upsetting or harming other individuals, advocates collective living-social harmony, SM, SP and risk perception increased while fatalistic tendencies decreased. Since conformity here is a social norm rather than a rule, it is thought to have a positive result in people's behaviours. Similarly, it was also found that the value of the *security-personal value*, which considers the safety of oneself and one's immediate environment, negatively affects fatalistic tendencies, but positively affects SM, SP and risk perception.

In this research, it has been determined that fatalistic tendencies are directly affected by values and it has been observed that fatalistic tendencies are quite high in Turk society.

Indeed, Acevedo [87] using data from the World Value Survey and the 2002 Gallup survey of Islamic countries, found that Türkiye, which has the longest western influence among Islamic countries, shows one of the highest levels of fatalism among Islamic countries. Causal attributions made by fatalists reflect a lack of control over risks [88].

Similar to this study, which found that SM directly affects SP, similar to this study, including in Türkiye [67], there are many studies including [51,57,[89], [90], [91]].

Another study found that security values are among the most important values in Mediterranean and Eastern European countries, but among the least important in American and Scandinavian countries. This probably reflects objective differences in the military, economic or personal threats to which people were exposed in previous decades [38]. This study also shows that security personal and security societal values are the two most important values in Türkiye.

## 5 Conclusion

Although national culture has featured more prominently on the safety research agenda in recent years ([92]; K. [78,93]) there is little research on the impact of current national culture on safety climate and safe/unsafe behaviours, and a framework that can guide our understanding of OHS and identify promising areas for research that advances the field is still lacking (K. [78,93]). This study attempts to fill this gap in the literature.

Learning the value dimensions of a community is important not only for OHS but also for psychology, sociology and many other disciplines. In this study, value dimensions and fatalistic tendencies of the Turk society were determined in detail according to NUTS-12 region. Considering that individuals will show behaviours according to the value dimensions they attach importance to, it is necessary to develop trainings in accordance with the value dimensions given importance according to NUTS-12 regions. Because if the use of language, methods and methods in education conflicts with the value judgments of the region, it will not be possible for the education to be successful. For example, it was found that conformity-rules negatively affected SP, whereas conformity-interpersonal value positively affected SP. We mentioned how this is supported by the theories of Trompenaars & Hampden-Turner [85]. It would be beneficial to use a language based on why it is important to avoid negativity or harm to other individuals in the event of a negative situation, as opposed to a rule-obligation of OHS measures applied in Turk society. Changing value judgments, which are the most fundamental of culture, is a phenomenon that takes time and even generations. For this reason, policy makers have a duty to eliminate and reduce the negative aspects of value judgments for OHS rather than changing the value dimensions.

This study focused on individual factors. Future studies may address individual and organizational factors together.

### Limitations of the study

The limitations of this study are that people over the age of 18 and working were selected by random sampling method.

## Declarations

Prof. Dr. Alim Yılmaz, Prof. Dr. Hayrunisa Alan, Prof. Dr. Tuncay Başoğlu, Prof. Dr. Özden Zeynep Oktav, Prof. Dr. Tarkan Oktay, Prof. Dr. Ahmet Akın and Prof. Dr. İbrahim Subaşı are the members of the ethics committee who approved the study.

## The informed consent statement

All participants provided informed consent to participate in the study.

### Data availability statement

Data will be made available on request.

## CRediT authorship contribution statement

**Ahmet Ebrar Sakalli:** Writing – review & editing, Writing – original draft, Methodology, Investigation, Data curation. **Selma Arikan:** Writing – review & editing, Supervision, Conceptualization.

## Declaration of competing interest

The authors declare that they have no known competing financial interests or personal relationships that could have appeared to influence the work reported in this paper.
